# Methylation of RAD51B, XRCC3 and other homologous recombination genes is associated with expression of immune checkpoints and an inflammatory signature in squamous cell carcinoma of the head and neck, lung and cervix

**DOI:** 10.18632/oncotarget.12211

**Published:** 2016-09-23

**Authors:** Damian T. Rieke, Sebastian Ochsenreither, Konrad Klinghammer, Tanguy Y. Seiwert, Frederick Klauschen, Inge Tinhofer, Ulrich Keilholz

**Affiliations:** ^1^ Charité Comprehensive Cancer Center, Charité – Universitätsmedizin Berlin, Berlin, Germany; ^2^ Department of Hematology and Medical Oncology, Campus Benjamin Franklin, Charité – Universitätsmedizin Berlin, Berlin, Germany; ^3^ Section of Hematology/Oncology, Department of Medicine, University of Chicago, Chicago, IL, USA; ^4^ Institute of Pathology, Charité - Universitätsmedizin Berlin, Berlin, Germany; ^5^ Department of Radiooncology and Radiotherapy, Charité – Universitätsmedizin Berlin, Berlin, Germany; ^6^ German Cancer Research Center Heidelberg (DKFZ)/German Cancer Consortium (DKTK) partner site Berlin, Berlin, Germany

**Keywords:** HRD, DNA repair, immune checkpoints, inflamed gene expression signature, immune therapy

## Abstract

Immune checkpoints are emerging treatment targets, but mechanisms underlying checkpoint expression are poorly understood. Since alterations in DNA repair genes have been connected to the efficacy of checkpoint inhibitors, we investigated associations between methylation of DNA repair genes and CTLA4 and CD274 (PD-L1) expression.

A list of DNA repair genes (179 genes) was selected from the literature, methylation status and expression of inflammation-associated genes (The Cancer Genome Atlas data) was correlated in head and neck squamous cell carcinoma (HNSCC), cervical and lung squamous cell carcinoma.

A significant positive correlation of the methylation status of 15, 3 and 2 genes with checkpoint expression was identified, respectively. RAD51B methylation was identified in all cancer subtypes. In HNSCC and cervical cancer, there was significant enrichment for homologous recombination genes. Methylation of the candidate genes was also associated with expression of other checkpoints, ligands, MHC- and T-cell associated genes as well as an interferon-inflammatory immune gene signature, predictive for the efficacy of PD-1 inhibition in HNSCC.

Homologous recombination deficiency might therefore be mediated by DNA repair gene hypermethylation and linked to an immune-evasive phenotype in SCC. The methylation status of these genes could represent a new predictive biomarker for immune checkpoint inhibition.

## INTRODUCTION

Cancer is a heterogeneous disease that is caused by alterations of the genome. Among these alterations, mutations account for a significant amount of the functional changes that have been identified to drive cancer. Many tumors also harbor a high mutational load with both driver and passenger mutations that accumulate through DNA damage, evolutionary selection and dysfunctional DNA repair. This also leads to a change in the structure of several intracellular proteins. Since proteins are degraded within the cell and also presented on MHC-1 proteins, the immune system is, in principle, able to recognize cancer cells. However, evasion from immune destruction has been recognized as one of the hallmarks of cancer [1]. Among the immunoevasive mechanisms, expression of immune checkpoint molecules and/or ligands that lead to T-cell inactivation has been increasingly recognized. Currently inhibitors of the immune checkpoints CTLA-4 (cytotoxic T-lymphocyte-associated protein 4) and CD274 (Programmed death-ligand 1, PD-L1 or B7H1) have been approved for the treatment of several cancer types, further underlining the importance of this pathway [2]. In head and neck squamous cell carcinoma, the overall response rate to pembrolizumab has been reported at 18% [3] and at 19.4% in NSCLC [4]. Several checkpoint inhibitors are in clinical trials for advanced squamous cell solid tumors, including cervical carcinoma (e.g. NCT01693783 [5], NCT01693562 [6] and others).

Many of the patients that respond to immunotherapy also exhibit durable responses. However, as noted above, only a fraction of all patients respond to checkpoint inhibitors. Robust predictors of a response have not yet been established in clinical routine. So far, expression of CD274 and CTLA4 have been shown to correlate with activity of checkpoint inhibitors. However, since cut-off values and staining patterns remain poorly defined and intratumoral heterogeneity is present, the therapeutic relevance of these biomarkers remains a matter of debate [7], [8].

In colorectal cancer, an immunoregulatory tumor environment and CD274 expression have been reported in microsatellite instable cancers (MSI) [9]. In a phase-II study, MSI was also associated with response to PD-1 blockade [10].

Microsatellite instability is caused by the inactivation of DNA enzymes in the mismatch excision repair pathway (MMR). In colorectal cancer, about 10% of cancers exhibit this phenotype. In other cancer types MSI has been identified but remains poorly defined, for instance in squamous cell carcinomas. Hypermethylation of MLH1 and MSH2 genes have been implicated as a potential cause of MSI in head and neck squamous cell carcinoma [11]. In ovarian cancer, alterations of homologous repair genes including BRCA1 have been associated with CD274 expression and tumor-infiltrating lymphocytes [12]. Homologous recombination is another DNA repair pathway that has been linked to the repair of DNA double strand breaks [13, 14]. RAD51B and XRCC3 are involved in homologous recombination, DNA sensing and apoptosis induction [15],[16],[17]. RAD51B and XRCC3 polymorphisms have been identified as a risk factor for prostate, ovarian, breast, head and neck and other cancer types [18],[19],[20]. In HNSCC, RAD51B is among the integration sites for HPV DNA, leading to an inactive form of the protein [21].

Further investigations into the connection between methylation of DNA repair genes and CD274 and immunoregulatory gene expression are lacking to date in squamous cell carcinomas. We therefore investigated associations between methylation of DNA repair genes and expression of the immune checkpoints CD274 and CTLA4.

## RESULTS

### Identification of a DNA repair gene methylation immune signature in squamous cell cancer types

In HNSCC, hypermethylation of 19 genes was identified to correlate with CD274 expression according to the defined cutoff (spearman correlation of 0.3). 16 genes were identified to correlate with CTLA4. 15 genes were present in both subsets and included in the HNSCC DNA repair gene candidate list (XRCC1, MLH3, PMS1, RAD51B, XRCC3, RAD54B, BRCA1, SHFM1, GEN1, FANCE, FAAP20, SPRTN, SETMAR, HUS1, and PER1).

In lung squamous cell carcinoma, 38 genes were identified for CTLA4, 20 genes for CD274 expression. 2 genes were present in both subsets and included in the lung squamous candidate list (RAD51B, CHEK1).

**Figure 1:**
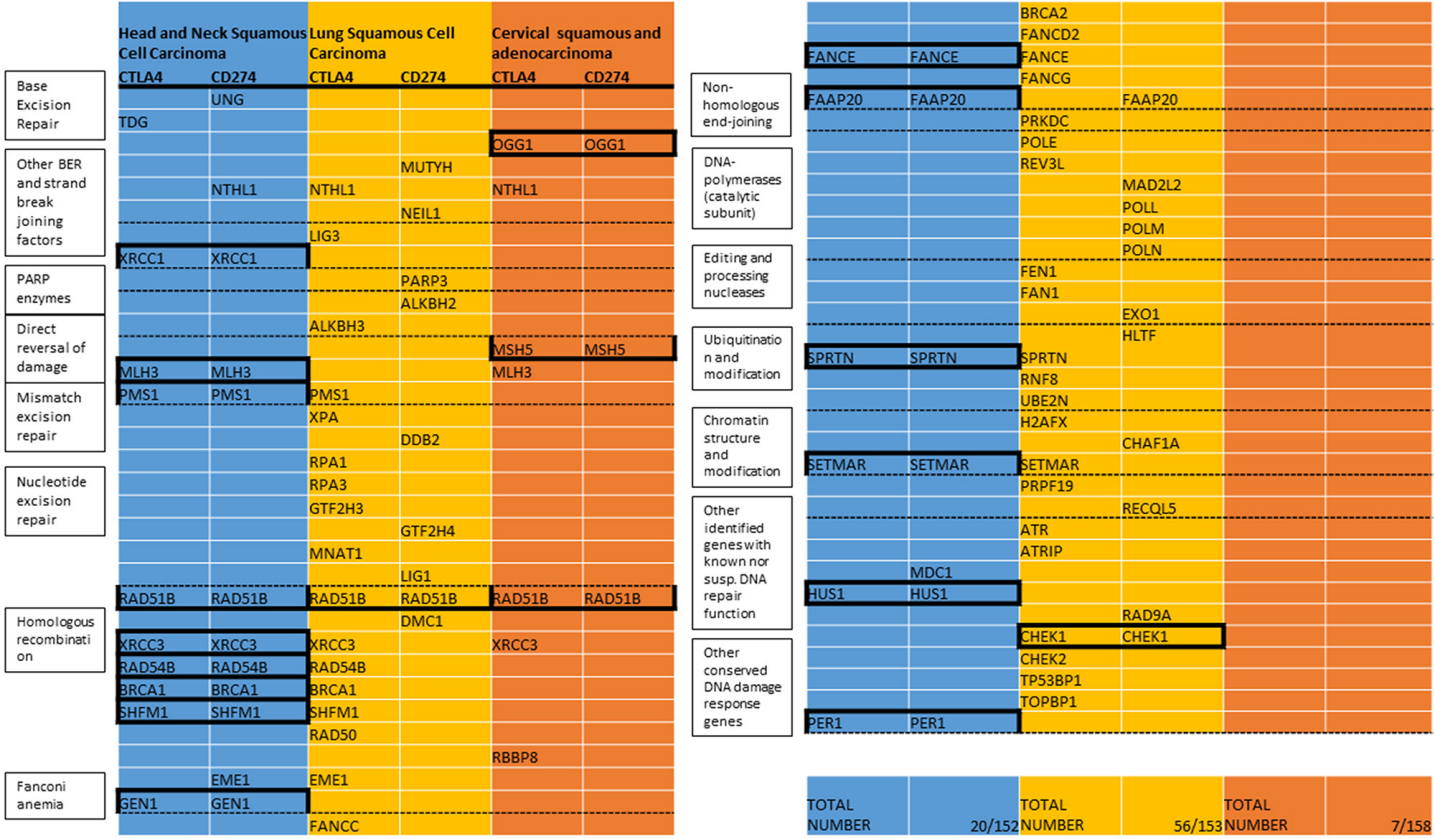
List of DNA repair genes with methylation associated with expression of CTLA4 or CD274 (TCGA) Included are all genes with correlation coefficients (spearman) exceeding 0.3 in the respective cancer types. Mechanistic groups are listed in the boxes corresponding to the dashed lines. The total number of repair genes in this list and number of all repair genes with available data in the respective types are listed under “Total Number”. Genes that exceed the threshold for CTLA4 and CD274 are included in the candidate gene list for the respective cancer type (indicated by black boxes). All Bonferroni corrected p-values for the candidate genes are < 0.05 (in the lung cancer candidate genes only one of the correlations remained significant after multiple testing correction, respectively).

In cervical cancer, 7 genes were identified for CTLA4, 3 genes for CD274. 3 genes were correlated with CTLA4 and CD274 and included in the cervical candidate list (OGG1, MSH5, and RAD51B). (Figure 1) The homologous recombination gene RAD51B was identified in all three candidate lists. Another homologous recombination gene, XRCC3, was identified to correlate with CTLA4 expression in all three cancer types but exceeded the cutoff for CD274 only in head and neck squamous cell carcinoma.

All Bonferroni corrected p-values for the candidate genes were < 0.05 with the exception of the two lung cancer candidate genes, where only one of the two correlations (RAD51B-CTLA4 and CHEK1-CD274) remained significant after correcting for multiple testing, respectively.

**Figure 2:**
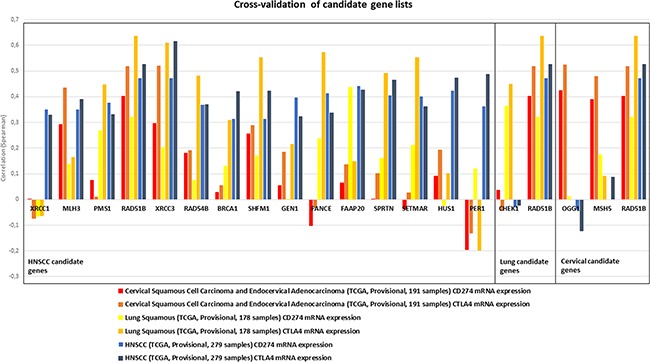
Candidate gene lists, as established for HNSCC (left box), lung squamous (middle box) and cervical carcinoma (right box) were cross-validated in the respective other cancer types for correlation with CD274 and CTLA4 expression (indicated by color) Several genes in the HNSCC candidate list showed similar correlation values also in lung and cervical cancer. MLH3, PMS1, RAD51B, XRCC3, RAD54B, BRCA1, SHFM1, FAAP20 and SPRTN exhibited positive correlations in all three cancer types.

No relevant negative correlations were observed across all genes. The established candidate gene lists were cross-validated in the respective other squamous cell carcinoma subtypes for the expression of CD274 and CTLA4. Most genes from the HNSCC list showed positive correlations with CTLA4 and CD274 expression in cervical and lung squamous cell carcinoma datasets with the homologous recombination genes XRCC3, RAD51B, RAD54B, SHFM1, and BRCA1, mismatch excision repair genes MLH3 and PMS1, fanconi anemia gene FAAP20 and a gene involved in ubiquitination and modification, SPRTN, exhibiting positive correlations in all tested associations (Figure 2). Cross-validation of lung squamous and cervical candidate lists identified only RAD51B to exhibit a relevant correlation with checkpoint expression in the respective other squamous cancer types.

### Patterns of methylation immune signatures vary between different cancer types

Two homologous recombination genes, X-Ray Repair Complementing Defective Repair in Chinese Hamster Cells 3 (XRCC3) and RAD51 Paralog B (RAD51B) exhibited the highest correlation with CD274 and CTLA4 across all three squamous cell cancer types.

**Figure 3:**
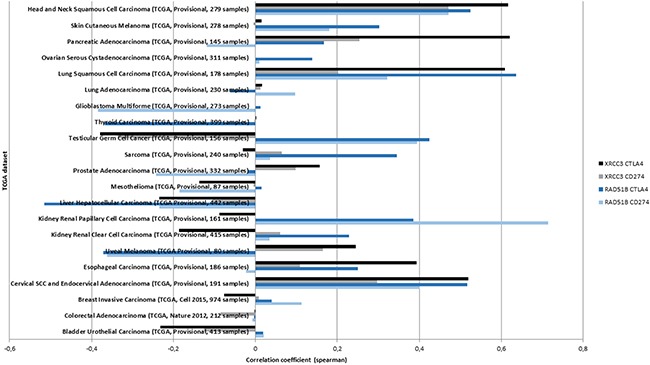
The correlation between XRCC3 and RAD51B methylation and mRNA expression of CD274 or CTLA4 is tested in available TCGA datasets (y-axis) The highest correlations (x-axis) across all four interactions were identified in the three squamous cancer histologies. Positive associations were not identified in all cancer types suggesting a histology-specific methylation signature.

To further validate a histology-dependent effect, RAD51B and XRCC3, and expression of CTLA4 and CD274 were evaluated in 21 TCGA datasets from cbioportal.org. Identified correlations varied substantially between the cancer types. Positive correlations in all four interactions were only identified for lung, cervical and head and neck squamous cancer histologies but several cancer types showed positive correlations for either RAD51B (e.g. kidney renal papillary cell carcinoma, testicular germ cell cancer) or XRCC3 (e.g. pancreatic adenocarcinoma) (Figure 3).

### Squamous cell candidate genes are differentially methylated

**Figure 4:**
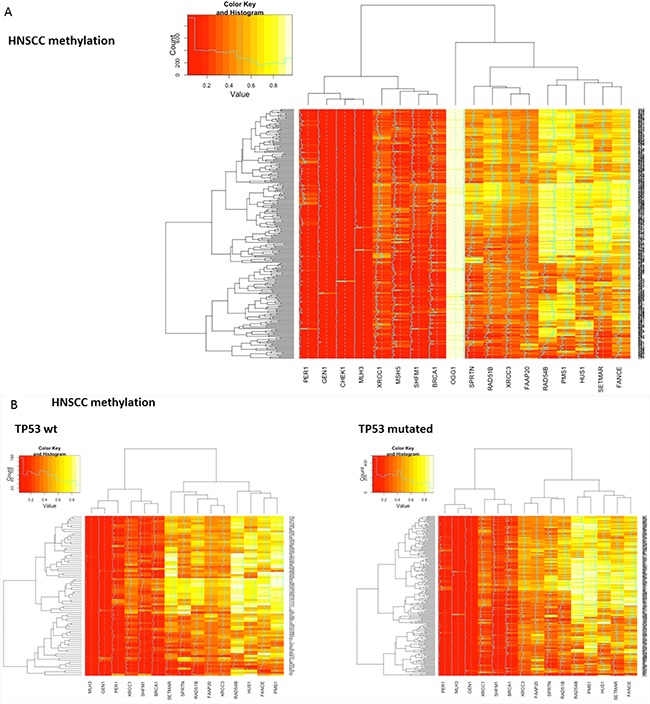

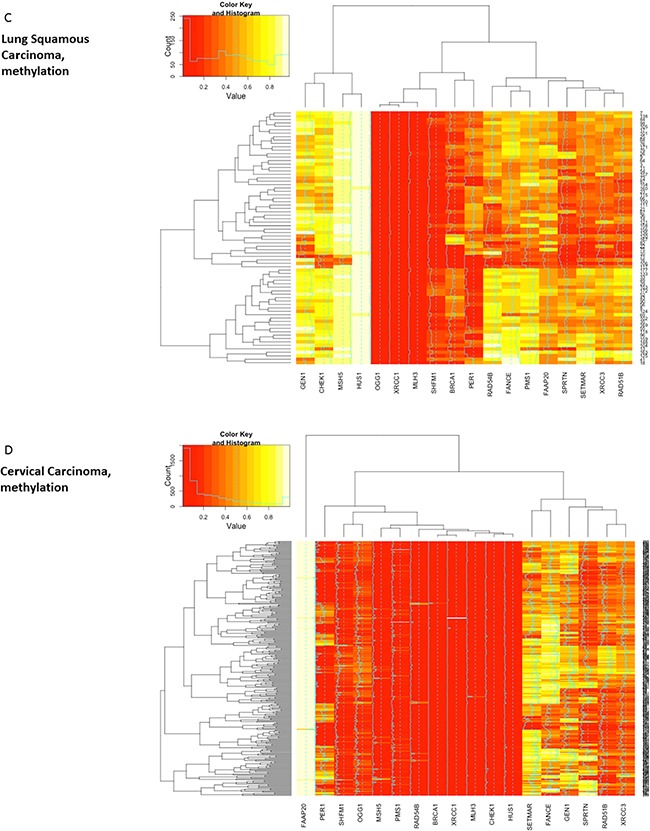
Heatmaps showing clustering and differential methylation of candidate genes from all three squamous histologies **A.** Heatmap for HNSCC. Among the genes with no or little differential methylation are all candidate genes from lung and cervical histology that were not identified in HNSCC (CHEK1, OGG1, CHEK1, MSH5). XRCC3, RAD51B and SPRTN that showed similar correlations across all three cancer types cluster closely together. **B.** No relevant differences are identified between TP53 mutant and TP53 wt samples as surrogate parameter for HPV status. **C.** Heatmap for lung squamous cell carcinoma. SPRTN, XRCC3 and RAD51B again exhibit differential methylation and cluster closely together. **D.** Heatmap for cervical carcinoma. Hypomethylation seems more prominent in most genes and RAD51B, SPRTN and XRCC3 again cluster closely together.

Next, patterns of methylation were investigated. Differential methylation data for all candidate DNA repair genes from all three squamous cell cancer types (N=18) were assessed. Most candidate DNA repair genes exhibited differential methylation in the respective cancer histology. Uniform hyper- or hypomethylation was mostly present in genes that showed little or no correlation in the cross-validation (e.g. OGG1 in HNSCC and LSCC, CHEK1 in HNSCC and cervical carcinoma, HUS1 in LSCC and cervical carcinoma, Figure 4). Among the DNA-repair genes that did exhibit positive correlations in the cross-validation, MLH3 was almost uniformly hypomethylated in all three cancer types. SHFM1 also showed high levels of hypomethylation in all three cancer types. PMS1, RAD54B, FAAP20 and BRCA1 exhibited almost uniform hyper- or hypomethylation in the cervical cancer samples. In contrast, RAD51B, XRCC3 and SPRTN were differentially methylated and also clustered closely together in all three cancer types. To assess methylation differences between biologic subtypes of HNSCC, the methylation signatures of TP53 mutant and TP53 wild type HNSCC were compared and did not show relevant differences (Figure 4b).

Further analyses with covariance and correlation matrices of candidate gene methylation status in HNSCC also identified the same differentially methylated genes. In these analyses, RAD51B was the most prominent gene (Supplementary Figure S4).

Methylation of the candidate DNA-repair genes was negatively correlated with mRNA expression of the respective gene (Supplementary Figure S5).

### Subgroup evaluation identifies the homologous recombination pathway

To analyze patterns of methylation in DNA repair genes, an in-silico evaluation was performed for the HNSCC DNA repair candidate genes using the predefined DNA repair mechanistic groups (Supplementary Figure S2).

Statistical analysis showed a significant enrichment in the homologous recombination pathway (percent candidate genes vs. percent total repair genes 40% vs. 12.5%, p=0.004). Since lung squamous and cervical cancer candidate gene lists encompassed not enough genes for statistical analysis, all genes that correlated with either CD274 or CTLA4 (compare Figure 1) were included in the analysis for these cancer types (cervical cancer N= 7, lung squamous N=56). Correlations of all 7 cervical carcinoma genes were statistically significant after Bonferroni correction but because of the small sample size, only 22 of the lung squamous cell carcinoma genes were significant after correcting for multiple testing (NTHL1, ALKBH2, ALKBH3, PMS1, GTF2H3, GTF2H4, MNAT1, RAD51B-CTLA4, XRCC3, RAD54B, SHFM1, EME1, FANCC, BRCA2, FANCE, FAAP20, PRKDC, REV3L, SPRTN, RNF8, SETMAR, CHEK1-CD274, TP53BP1).

**Figure 5:**
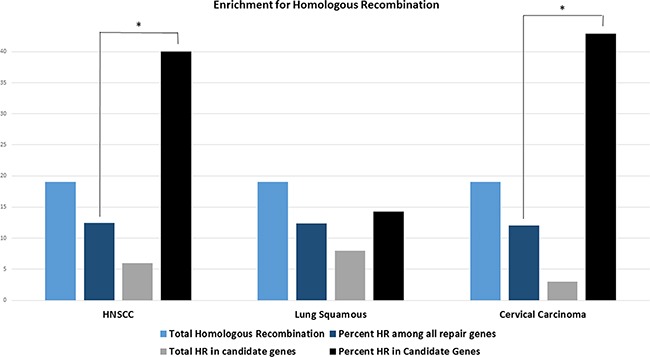
Bars indicate the total number of homologous recombination (HR) genes across all DNA repair genes (light blue), the total number of HR genes in the respective DNA repair candidate genes (grey), the percentage of HR genes among all repair genes (dark blue) and the percentage of HR genes in all DNA repair candidate genes (black) in the respective cancer types HNSCC candidate genes show statistically significant enrichment for the homologous recombination pathway (p=0.004, one-sided student's t-test). All genes that exceed the threshold for either CTLA4 or CD274 were included for the analysis in cervical and lung squamous histologies since candidate genes were too few for statistical analysis in both cancer types. All cervical carcinoma genes but only 22 lung squamous cell carcinoma genes were statistically significant after correcting for multiple testing. Cervical carcinoma also shows a statistically significant enrichment for the homologous recombination pathway (p=0.038). Asterisk indicating p<0.05.

Genes involved in homologous recombination also showed statistically significant (p=0.038) enrichment in cervical cancer (Figure 5). Genes involved in homologous recombination and nucleotide excision repair pathways made up the highest absolute (n=8) number of genes in lung squamous cell carcinoma but did not reach statistical significance for relative enrichment also when only the 22 genes with statistical significance were considered (p=0.36).

### Methylation of candidate genes is associated with a predictive inflammatory signature

**Figure 6:**
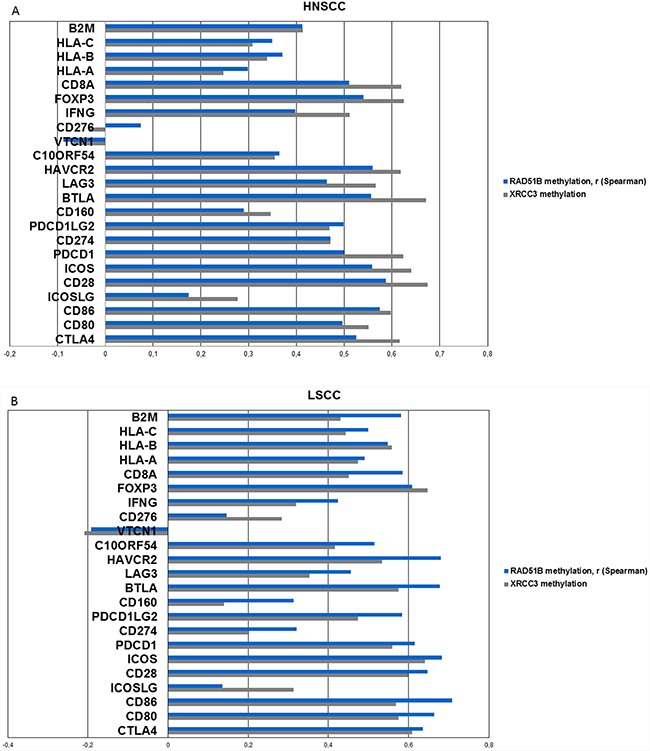

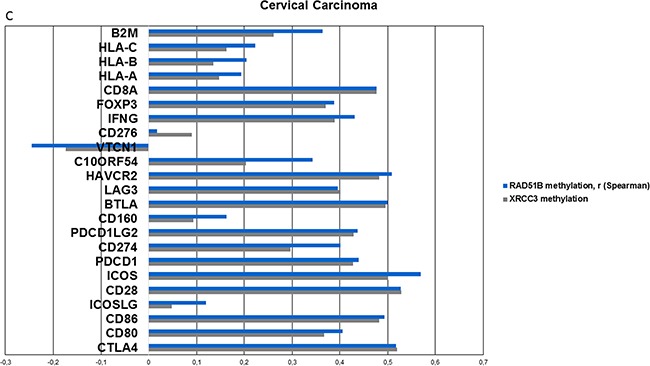
Methylation of candidate genes is analyzed for correlation with mRNA expression of a broad range of inflammation-associated genes (including checkpoint molecules, ligands, T-cell markers, interferon gamma, MHC1 and 2, TCGA) **A.** Correlations (spearman) of the two top-performing genes from previous analyses, RAD51B and XRCC3 with inflammation-associated genes in HNSCC. Positive correlations are identified in all genes except for CD276 and VTCN1. All Bonferroni corrected p-values < 0.001 with the exception of CD276, VTCN1 and ICOSLG-RAD51B interaction (p>0.5). **B.** Correlation values (spearman) of XRCC3 and RAD51B in lung squamous cell carcinoma exhibits a similar pattern like HNSCC. **C.** Correlations (spearman) of XRCC3 and RAD51B in cervical carcinoma. CD276 and VTCN1 are again the only outliers and VTCN1 shows a statistically significant negative correlation with RAD51B (p=0.015) methylation after Bonferroni correction.

Since the two homologous recombination genes XRCC3 and RAD51B were identified in the cross-validation and also found to be differentially methylated in all cancer types, they were further analyzed for correlations with a broad panel of immune-associated genes, including checkpoint molecules, checkpoint ligands, interferon gamma, T-cell markers and HLA-1 associated genes (Figure 6, Supplementary Figure S3 and S7).

Methylation of XRCC3 and RAD51B exhibited statistically significant (all Bonferroni corrected p-values <0.001, with the exception of CD276, VTCN1 and ICOSLG-RAD51B interaction), strong positive correlations with expression of inflammation associated genes in head and neck squamous cell carcinoma and also showed similar patterns in lung and cervical carcinoma (Figure 6a, 6b, 6c). Two out of the 23 tested genes showed less prominent or negative correlation values. These outliers (CD276, VTCN1) were present in all three cancer subtypes (Figure 6). The same was found when the DNA repair candidate genes from cervical carcinoma (MSH5, OGG1) and lung squamous cell carcinoma (CHEK1) were analyzed (Supplementary Figure S12a, S12b). In cervical carcinoma, VTCN1 exhibited a negative correlation with OGG1 and RAD51B methylation that was statistically significant after Bonferroni correction (p=0.009, p=0.015, respectively).

**Figure 7:**
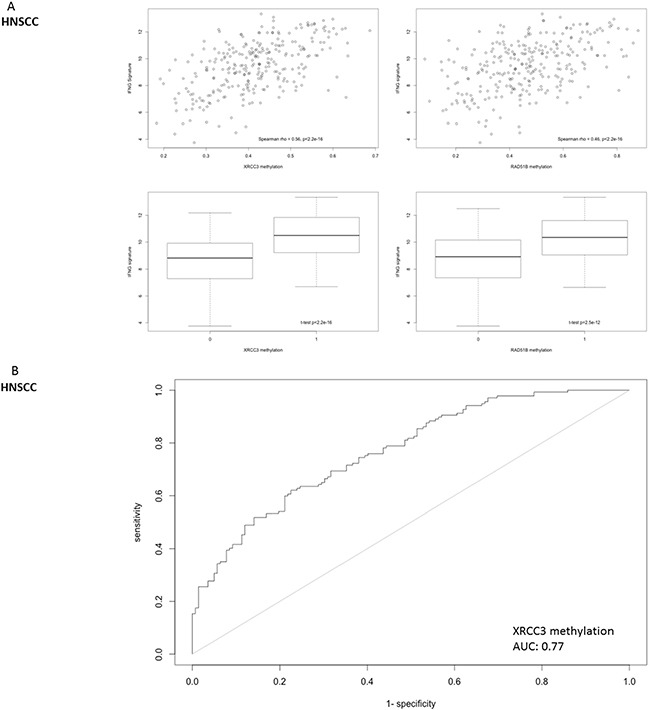

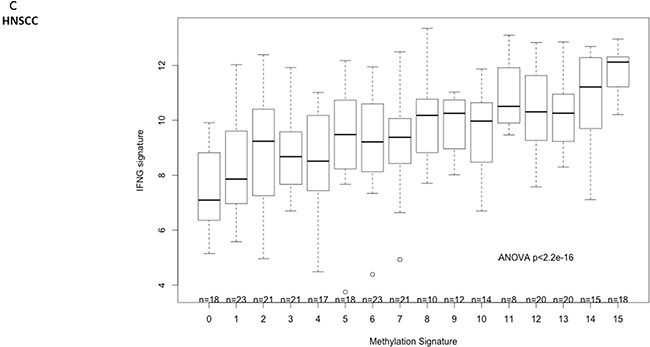
Correlation of DNA repair gene methylation with the expression of a 6-gene IFNG signature (consisting of STAT1, HLA-DRA, IFNG, IDO1, CXCL9 and CXCL10) that has been identified as a predictive biomarker for PD-1 inhibitor efficacy in head and neck cancer **A.** Methylation of RAD51B and XRCC3 is correlated with the six-gene IFNG-signature expression in HNSCC. **B.** The ROC-area under the curve for the predictive value of XRCC3 binomial methylation (as defined by methylation > mean) is 0.77. **C.** The IFNG-signature shows a dose-dependent expression according to the number of hypermethylated candidate genes in HNSCC (methylation signature. A score of 1 indicates one candidate DNA repair gene with methylation > mean).

To further analyze clinical relevance, a six-gene “interferon-inflammatory immune gene signature” (IFNG signature, consisting of genes IFNG, HLA-DRA, CXCL9, CXCL10, IDO1 and STAT1) that has been identified as predictive for patient response to checkpoint inhibitors in melanoma and HNSCC, was interrogated in HNSCC [3],[22]. This inflammation signature was significantly correlated with XRCC3 and RAD51B gene methylation (Figure 7a). An area under the curve of the receiver operating characteristic curve of 0.77 was reached with binary XRCC3 methylation for this signature (Figure 7b). Expression of the IFNG signature was not only correlated to methylation on the single gene level, but also significantly higher in patients with hypermethylation of more candidate genes in a dose-dependent manner (methylation signature) (Figure 7c).

### Mutation of DNA repair genes is not correlated with CD274 expression

We next assessed if mutations in DNA repair genes are also involved in tumor inflammation in HNSCC. Mutations of genes involved in DNA repair pathways were present in almost 88.5% of the samples (TP53 mutations included, Supplementary Figure S10). However, the number of mutations in all DNA repair genes was not correlated to the expression of CD274 (spearman rho: -0.08, p=0.2, Supplementary Figure S8a). The same was found when only mutations in genes from the HNSCC candidate gene list were included (9.3% of cases with at least one mutation, rho: -0.07, p=0.27, Supplementary Figure S8b). In contrast, the methylation signature, as defined by methylation of every gene from the HNSCC candidate gene list was significantly correlated with CD274 mRNA expression (93.5% of cases with at least one gene hypermethylated, rho: 0.53, p<0.001, Supplementary Figure S8d). When methylation and total mutation scores were combined, only one sample (1/279) exhibited, neither hypermethylation in the candidate gene list, nor mutations among all DNA repair genes. This patient sample did not show relevant CD274 expression. As expected, the correlation of the combined score (methylation and mutation) with CD274 expression was less pronounced than for the methylation score alone (Supplementary Figure S8c).

In addition to the mutations of DNA repair genes, the total mutational load in HNSCC tumor samples was assessed, since a nonfunctional DNA repair mechanisms might lead to an increase in mutations and thus lead to a tumor inflammation. However, the mutational load did not differ with respect to XRCC3 methylation, and was also unrelated to the IFNG-signature (Supplementary Figure S9).

## DISCUSSION

This report for the first time proposes a link between hypermethylation of DNA-repair genes and the expression of immune checkpoints in squamous cell carcinomas.

We systematically searched for methylation of DNA repair genes associated with CD274 and CTLA4 expression. We chose these two genes to represent both a targetable alteration on the tumor cell (CD274) as well as a targetable indicator of a tumor immune microenvironment (CTLA4). We identified genes from the homologous recombination pathway in the gene lists and cross-validation of all three candidate gene sets revealed striking overlaps between the different cancer types with several genes including XRCC3 and RAD51B as potential candidates for further investigation. Methylation and mRNA expression were negatively correlated (Supplementary Figure S6), which is not surprising since cbioportal.org predefines those DNA methylation sites with the highest negative correlation to mRNA expression. However, the respective methylation sites of candidate genes were almost identical across tumor types (data not shown).

XRCC3 and RAD51B both are RAD51 paralogs that are involved in DNA double strand break repair [17], [15]. Homologous recombination deficiency (HRD) has also been shown in HNSCC and deficiency in RAD51 foci formation has been observed in HNSCC cell lines sensitive to PARP inhibitor rucaparib [23].

The here presented results suggest that in head and neck cancer HRD might be mediated by epigenetic silencing. In addition to the potential effects on immune checkpoint inhibition, HRD has been shown to predict sensitivity to treatment with PARP inhibitors and chemotherapeutic agents including platinum [24], [23], [15].

To assess the cause of the immune infiltration and expression of immune checkpoints, we also assessed the mutational load. The total number of mutations did not correlate with the methylation status of candidate genes but importantly it was also not associated with the interferon gamma signature. This result is in accordance with another report that did not find an association between an inflammatory microenvironment and total mutational load [25]. The creation of neoantigens through defective DNA repair might still play a role and has been identified to correlate with response to immunotherapy [26], [27].

Since the presented data are purely correlative, there are several options apart from mutational load for potential mechanisms between candidate DNA repair gene methylation and immune checkpoint expression.

It is possibly, albeit unlikely, that hypermethylation of these genes directly leads to pathway activation and upregulation of checkpoint molecules and inflammation.

As another possible explanation the observed methylation differences might represent the immune infiltrate rather than differences in the tumor. Tumor purity has been identified as a confounder of TCGA analyses [28]. However, a potential predictive value of the here described methylation pattern would not be affected by this, because the predictive interferon-inflammatory immune gene signatures largely consist of T-cell associated genes as well [3],[22]. Additionally, the differences in methylation patterns of candidate genes in other cancer histologies makes it likely that we observe a tumor-specific methylation pattern rather than a signature of the immune infiltrate.

Thirdly, it is possible that the methylation of the candidate genes represent a global methylation pattern observed in dedifferentiated cells associated with inflammation. Different gene methylation subgroups have been described in the TCGA HNSCC analysis, that also correlated with gene expression signatures [29].

The negative correlations between methylation of some candidate genes and immune checkpoint expression in some cancer types further underline a histology-dependent effect. Biological changes within a tumor might lead to dedifferentiation and methylation changes. At the same time, methylation changes might also be causal to histological dedifferentiation like an Epithelial-to-Mesenchymal Transition (EMT) [30]. EMT has accordingly been described in inflammatory lung cancers [25]. Additional experiments showed that methylation of XRCC3 and RAD51B is significantly correlated to the expression of the mesenchymal marker Vimentin and reduced expression of the epithelial marker E-Cadherin (Supplementary Figure S11A, S11B). Similar associations were also identified in lung and cervical carcinoma (data not shown).

In HNSCC, an EMT-associated expression subgroup was overlapping with an immune infiltrate and inflammation [31]. This expression subgroup was present in HPV positive and negative tumors. We also did not find differences in methylation between TP53 mutant and wild-type tumors, that can also serve as a surrogate marker of HPV infection.

The identification of an inflamed expression phenotype as linked with the candidate genes further supports the validity of these results. The identified genes comprise of further checkpoint proteins, ligands, as well as T-cell associated genes including interferon gamma. VTCN1 and CD276 form a distinct subgroup among the B7 receptor family. While the first subgroup of the B7 family, CD80, CD86 and ICOS-L, are stimulatory molecules interacting with the second subgroup of the B7 family, CD274 and CD273, which are inducible inhibitory receptors, or with CD28, the role in immunomodulation and oncogenicity of CD276 and VTCN1 is less clear and partly contradictory. For both, the ligand is not yet identified, they are expressed in non-hematopoietic tissues, they seem to act inhibitory on T-cells, and expression is negatively correlated to outcome in several tumor entities. [32], [33] Our experiments show not only that VTCN1 (B7-H4) and CD276 (B7-H3) did not show a positive correlation in all three squamous cancer types, but that CD276 is negatively correlated to CD8A expression in HNSCC (data not shown). This observation supports the immunosuppressive role predescribed for VTCN1 and CD276 and the special role they play among B7 checkpoint molecules.

This is further supported by another study in esophageal squamous cell carcinoma where expression of both genes was negatively correlated with the infiltration of CD8+ T-cells [34].

The interferon-inflammatory immune gene signature has been reported as predictive of response to pembrolizumab in HNSCC, gastric cancer and melanoma and as correlated to an immune-evasive phenotype in mesothelioma [3], [35], [22], [36]. The six-gene IFNG signature has been reported as predictive for pembrolizumab efficacy in HNSCC and was correlated with hypermethylation of the candidate genes in this study [3]. Importantly, this was found for candidate genes as well as for a multi-gene methylation signature thus proposing a dose-dependent effect on both the intra- as well as intergenic level.

An effect of hypomethylating agents on the increased expression of immune-related genes has recently been described [37]. The reactivation of endogenous retroviruses has been proposed as a potential mechanism of hypomethylating agents [38]. A rapid reactivation of retroviruses is expected shortly after introducing hypomethylating agents and the here proposed mechanism is expected to arise much later. Therefore these results are not conflicting with those reported here, but further suggest that there is no direct effect between hypermethylation of the DNA repair candidate genes (e.g. XRCC3, RAD51B) and expression of checkpoints. We therefore conclude that hypermethylation of homologous recombination DNA repair genes including RAD51B and XRCC3 is associated with an inflamed phenotype in squamous cell cancers of the head and neck, lung and cervix. These genes might represent promising markers for the expression of immune checkpoints and an inflamed phenotype and warrant further investigation, either alone or in combination with other genes, as predictive biomarkers for response to immunotherapy.

## MATERIALS AND METHODS

### Data

Publicly accessible data from the cancer genome atlas research network (TCGA) were used for HNSCC (TCGA provisional, 278 samples), cervical squamous and adenocarcinoma (TCGA provisional, 191 samples) and squamous cell carcinoma (TCGA provisional, 178 samples, methylation data available for 74 samples) and downloaded from cbioportal.org (01-29-2016) [29], [39], [40], [41]. Restrictions for the use of these data in publications were queried from http://cancergenome.nih.gov/publications/publicationguidelines, no restrictions were identified. Methylation data (HM450) were available for 152, 153 and 158 of the 179 DNA repair genes, respectively and downloaded/analyzed with mRNA expression data (RNA Seq V2 RSEM) for CTLA4, CD274 and other immune-associated genes as well as protein expression levels of CDH1 (reverse phase protein array, RPPA) from/in cbioportal.org.

### Statistical analysis

Correlation analyses and primary data visualization was performed in cbioportal.org. Validation and statistical analysis and visualization were performed in R (R: A Language and Environment for Statistical Computing, R Core Team, R Foundation for Statistical Computing, Vienna, Austria, 2015, https://www.R-project.org), using the following packages: Hmisc, Frank E Harrell Jr. et. al., http://biostat.mc.vanderbilt.edu/Hmisc; Scatterplot3d - an R Package for Visualizing Multivariate Data, Uwe Ligges and Martin Maechler, Journal of Statistical Software, 2003, pages 1-20, number = 11, volume = 8, url = http://www.jstatsoft.org; RGL - 3D Visualization Using OpenGL, Daniel Adler, Duncan Murdoch, https://r-forge.r-project.org/projects/rgl/; gplots: Various R Programming Tools for Plotting Data, Gregory R. Warnes, Ben Bolker, Lodewijk Bonebakker, Robert Gentleman, Wolfgang Huber Andy Liaw, Thomas Lumley, Martin Maechler, Arni Magnusson, Steffen Moeller, Marc Schwartz, Bill Venables, gplots_2.17.0.tar.gz; AUC-Threshold independent performance measures for probabilistic classifiers, Michel Ballings and Dirk Van den Poel.

### Establishment of candidate genes associated with an immune regulatory microenvironment

A list of 179 DNA repair genes was established from the literature (Supplementary Figure S2). (http://sciencepark.mdanderson.org/labs/wood/dna_repair_genes.html#MMR, updated from [42]).

Absolute DNA repair gene methylation status (HM450, TCGA data) was correlated with mRNA (RNAseq) expression of both, CD274 and CTLA4. DNA repair genes with rho (spearman) values exceeding the cutoff of rho=0.3 for CD274 and CTLA4 were included in the candidate gene lists for every squamous cancer type (HNSCC, lung, cervical carcinoma).

The cutoff > 0.3 was defined in HNSCC to encompass 10% of DNA repair genes with available data in HNSCC where the primary analysis was performed. A flow-chart showing the process of identification of candidate genes is available in the supplementary figures to this manuscript (Supplementary Figure S1).

Established gene-lists were cross-validated in the other squamous cancer types. P-values were adjusted according to the Bonferroni correction. Bonferroni corrected p-values < 0.05 were considered significant.

### Identification of the involved DNA repair pathways

DNA-repair pathways were defined according to the literature (compare above, Supplementary Figure S2). The HNSCC candidate gene list was analyzed for enrichment in DNA repair pathways by statistical evaluation of the group with the highest relative proportion of candidate genes (one-sided Fisher's exact test). Since there were too few genes in lung and cervical carcinoma candidate gene lists, all genes that exceeded the cutoff of 0.3 for either CTLA4 or CD274 (56 and 7 genes, respectively) were included for the enrichment analysis in these cancer types. Among the 56 genes from lung squamous cell carcinoma, only 22 were statistically significant after Bonferroni correction.

### Expression analysis of immune-related genes

A list of genes associated with an activated immune environment was established (Supplementary Figure S3). Correlation analysis was performed with the top-performing genes from the respective candidate gene list in the three squamous cell cancer types. P-values were adjusted according to the Bonferroni correction, Bonferroni p-values < 0.05 were considered significant. A six-gene interferon gamma inflammatory gene expression signature (IFNG signature) was derived from the literature [22], [3]. The mean log2 expression value of the sum of all six genes was used as IFNG expression signature. To derive a methylation signature, binary methylation status (0 for methylation <= mean methylation in the same gene, 1 for methylation > mean) of all candidate genes was assessed and added up to a methylation score. For the ROC-curve analysis, binary methylation status of candidate genes was defined by the same method (0 for methylation <= mean methylation in the same gene, 1 for methylation > mean).

### Mutation analysis

Mutational data were downloaded for all previously established DNA repair genes from cbioportal.org (TCGA data)(Supplementary Figure S2). To derive a mutation signature, mutation status of all DNA repair genes or candidate genes was assessed and +1 added to a score for every gene with at least one mutation. The total mutational load was downloaded from cbioportal.org (TCGA data).

## SUPPLEMENTARY FIGURES


