# Supplementary material for: Methylation of RAD51B, XRCC3 and other homologous recombination genes is associated with expression of immune checkpoints and an inflammatory signature in squamous cell carcinoma of the head and neck, lung and cervix
Source: Oncotarget. 2016 Sep 23;7(46):75379–93. doi: 10.18632/oncotarget.12211 (PMC5342748; doi:10.18632/oncotarget.12211)
Supplement: Supplementary file 1 [file oncotarget-07-75379-s001.pdf]

Methylation of RAD51B, XRCC3 and other homologous recombination genes is associated with expression of immune checkpoints and an inflammatory signature in squamous cell carcinoma of the head and neck, lung and cervix

SUPPLEMENTARY FIGURES

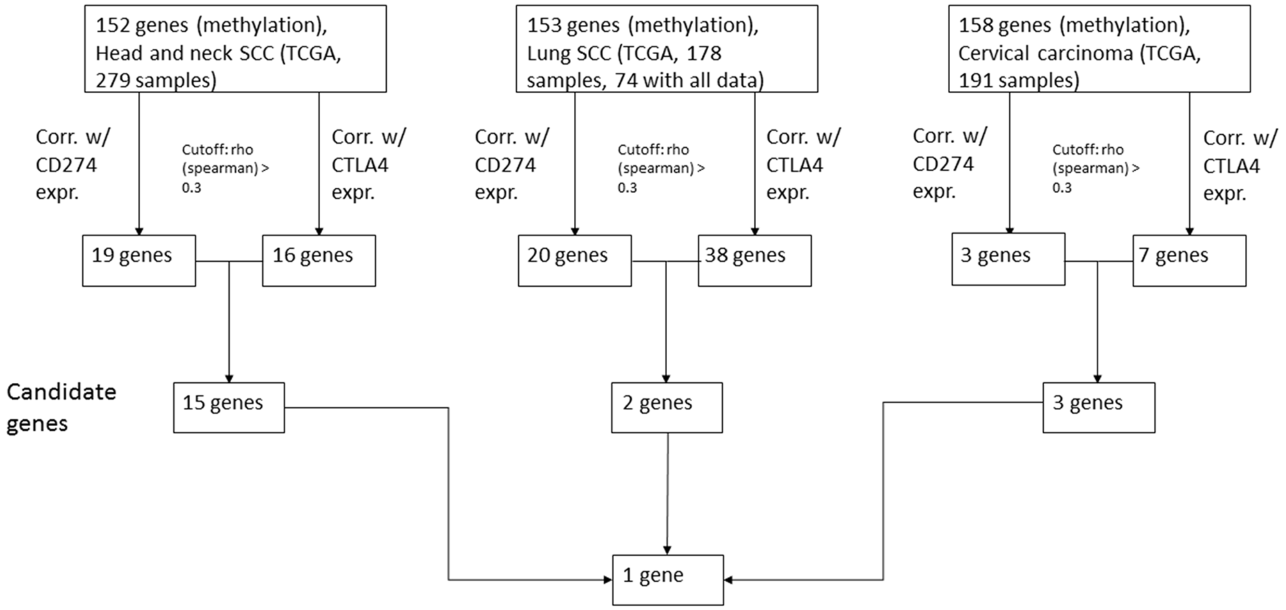

Supplementary Figure S1: Flow chart showing the establishment of candidate genes. Briefly, the methylation status of a list of DNA repair genes was correlated with CD274 and CTLA4 expression. Genes that exceeded the threshold of a spearman correlation coefficient of 0.3 for both immune checkpoints (CTLA4 and CD274) were included in the candidate gene list. One gene was identified in all three cancer types.

Base excision repair (BER)
UNG; SMUG1; MBD4; TDG; OGG1; MUTYH; NTHL1; MPG; NEIL1; NEIL2; NEIL3;

Other BER and strand break joining factors
APEX1; APEX2; LIG3; XRCC1; PNKP; APLF;

Poly(ADP-ribose) polymerase (PARP) enzymes that bind to DNA
PARP1; PARP2; PARP3;

Direct reversal of damage
MGMT; ALKBH2; ALKBH3;

Repair of DNA-topoisomerase crosslinks
TDP1; TDP2;

Mismatch excision repair (MMR)
MSH2; MSH3; MSH6; MLH1; PMS2; MSH4; MSH5; MLH3; PMS1; PMS2L3;

Nucleotide excision repair (NER)
XPC; RAD23B; CETN2; RAD23A; XPA; DDB1; DDB2; RPA1; RPA2; RPA3; TFIIH; ERCC3; ERCC2; GTF2H1; GTF2H2; GTF2H3; GTF2H4; GTF2H5;
CDK7; CCNH; MNAT1; ERCC5; ERCC1; ERCC4; LIG1; ERCC8; ERCC6; UVSSA; XAB2; MMS19;

Homologous recombination
RAD51; RAD51B; RAD51D; DMC1; XRCC2; XRCC3; RAD52; RAD54L; RAD54B; BRCA1; SHFM1; RAD50; MRE11A; NBN; RBBP8; MUS81; EME1;
EME2; GIYD1; GIYD2; GEN1;

Fanconi anemia
FANCA; FANCB; FANCC; BRCA2; FANCD2; FANCE; FANCF; FANCG; FANCI; BRIP1; FANCL; FANCM; PALB2; RAD51C; BTBD12; FAAP20; FAP24;

Non-homologous end-joining
XRCC6; XRCC5; PRKDC; LIG4; XRCC4; DCLRE1C; NHEJ1;

Modulation of nucleotide pools
NUDT1; DUT; RRM2B;

DNA polymerases (catalytic subunits)
POLB; POLG; POLD1; POLE; PCNA; REV3L; MAD2L2; REV1L; POLH; POLI; POLQ; POLK; POLL; POLM; POLN;

Editing and processing nucleases
FEN1; FAN1; TREX1; TREX2; EXO1; APTX; SPO11; ENDOV;

Ubiquitination and modification
UBE2A; UBE2B; RAD18; SHPRH; HLTF; RNF168; SPRTN; RNF8; RNF4; UBE2V2; UBE2N;

Chromatin Structure and Modification
H2AFX; CHAF1A; SETMAR;

Genes defective in diseases associated with sensitivity to DNA damaging agents
BLM; WRN; RECQL4; ATM; TTDN1;

Other identified genes with known or suspected DNA repair function
DCLRE1A; DCLRE1B; RPA4;; PRPF19; RECQL; RECQL5; HELQ; RDM1; OBFC2B;

Other conserved DNA damage response genes
ATR; ATRIP; MDC1; RAD1; RAD9A; HUS1; RAD17; CHEK1; CHEK2; TP53; TP53BP1; RIF1; TOPBP1; CLK2; PER1

source: http://sciencepark.mdanderson.org/labs/wood/dna_repair_genes.html#MMR

Supplementary Figure S2: DNA repair gene list as established from the literature. Source is mentioned below.

MHC-1 marker:
B2M; HLA-A; HLA-B; HLA-C

T-cell marker
CD8A; FOXP3; CD160; CTLA4

Interferon gamma
IFNG

Immune checkpoint molecules
CD276; C10ORF54; HAVCR2; LAG3; BTLA; PDCD1; ICOS; CD28; CTLA4;

Checkpoint ligands
PDCD1LG2; CD274; ICOSLG; CD86; CD80;

Costimulatory proteins
VTCN1

Supplementary Figure S3: List of genes associated with an activated immune environment.

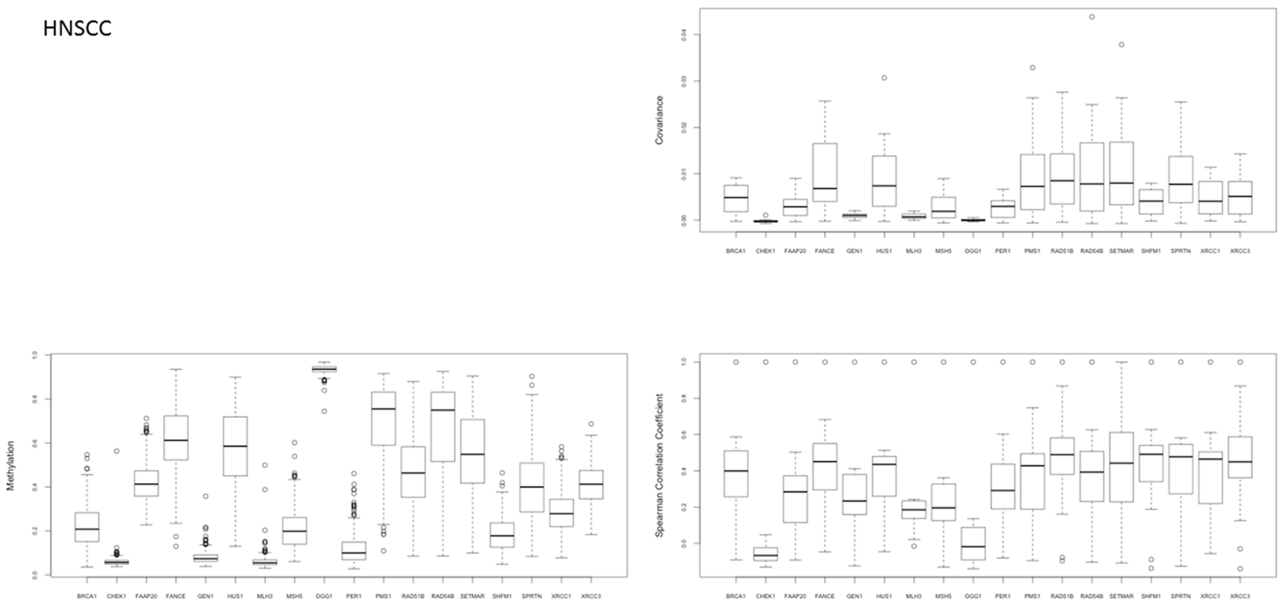

Supplementary Figure S4: Boxplots of differential methylation, covariance and correlation coefficients of DNA-repair gene methylation in HNSCC (TCGA). Genes from lung and cervical carcinoma candidate gene lists are identified as outliers.

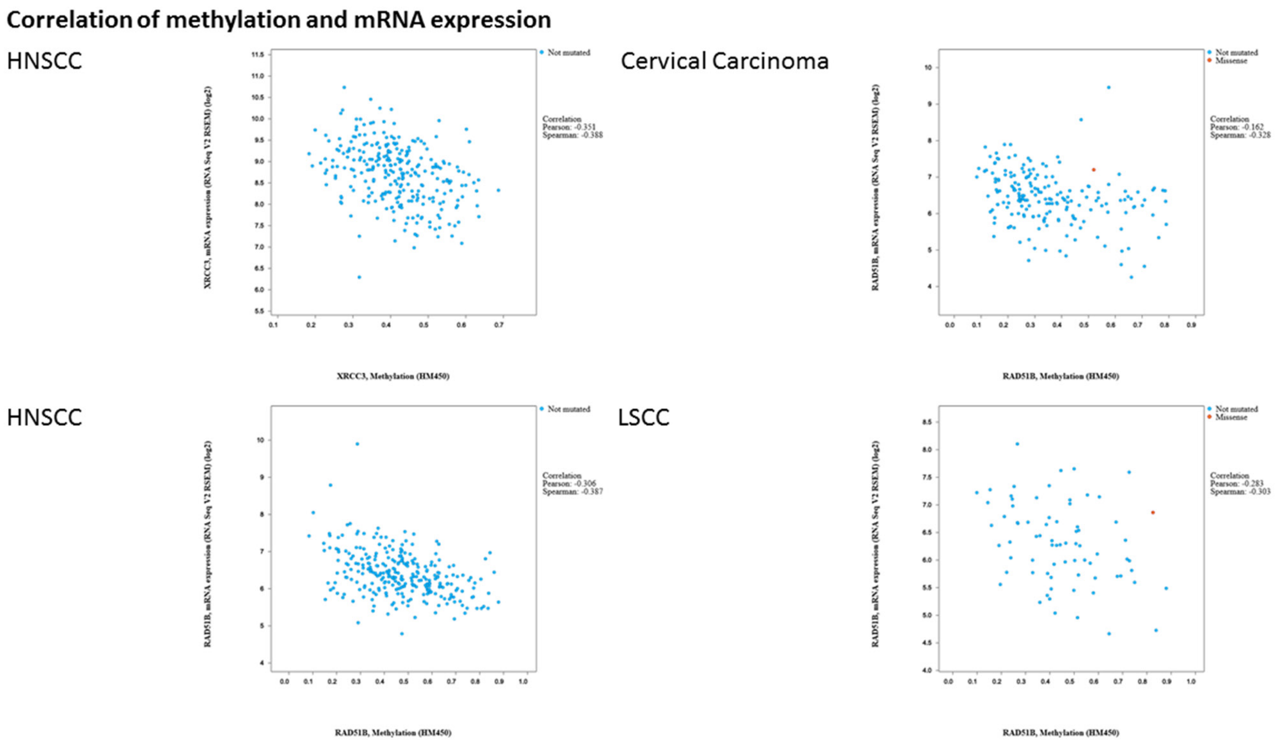

Supplementary Figure S5: Methylation of XRCC3 (HNSCC) and RAD51B (HNSCC, lung, cervical) is negatively correlated with expression in the TCGA dataset. The top left panel shows XRCC3 methylation (x-axis) and expression (y-axis) correlation. The spearman correlation coefficient is -0.388. Bottom left panel shows RAD51B methylation and expression, the spearman correlation is -0.387. The right panels show RAD51B methylation and expression correlation in cervical carcinoma (top right, spearman correlation coefficient -0.328) and lung squamous cell carcinoma (LSCC, bottom right, spearman rho= -0.303).

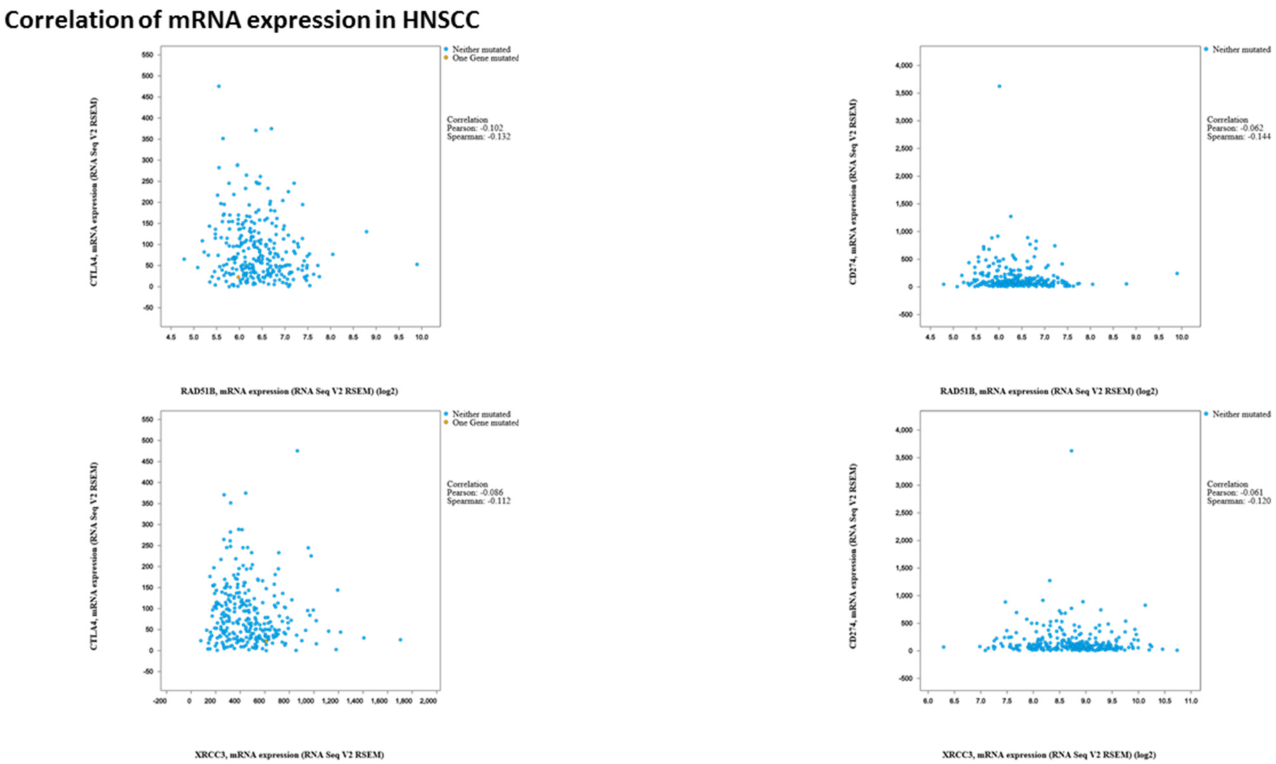

Supplementary Figure S6: Correlation of mRNA expression values of RAD51B and XRCC3 and mRNA immune checkpoint expression in HNSCC. Expression of RAD51B and XRCC3 is negatively correlated with the expression of CTLA4 and CD274. The top left panel shows the correlation between RAD51B mRNA expression (log2, x-axis) and CTLA4 expression (y-axis). The spearman correlation coefficient is -0.132. Bottom left panel shows the correlation between XRCC3 mRNA expression (x-axis) and CTLA4 mRNA expression (y-axis) with a spearman rho of -0.112. The right panels show the correlation between RAD51B mRNA expression (top panel, rho = -0.144) and XRCC3 mRNA expression (bottom panel, rho=-0.120) and CD274 expression.

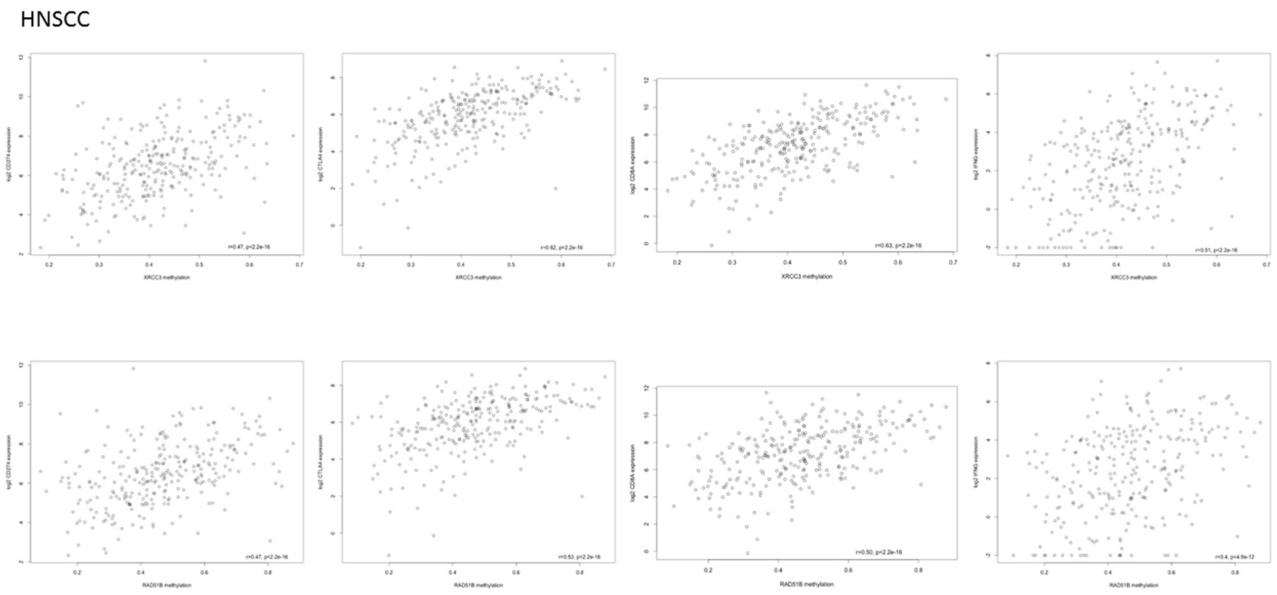

Supplementary Figure S7: Scatterplots of the correlations between XRCC3 and RAD51B methylation and expression of CTLA4, CD274 and Interferon gamma in HNSCC (TCGA). Correlation coefficients (spearman) and p-values are shown in the scatterplots. The top panels show the correlation between XRCC3 methylation (x-axis) and log2 CD274 (left, r=0.47, p<2.2e-16), CTLA4 (middle left, r=0.62, p<2.2e-16), CD8A (middle right, r=0.63, p=2.2e-16), and IFNG expression (right, r=0.51, p<2.2e-16). The bottom panels show the correlation between RAD51B methylation and the respective gene expressions (from left to right: CD274, r=0.47, p<2.2e-16, CTLA4, r=0.53, p<2.2e-16, CD8A, r=0.5, p<2.2e-16, IFNG, r=0.4, p=4.9e-12).

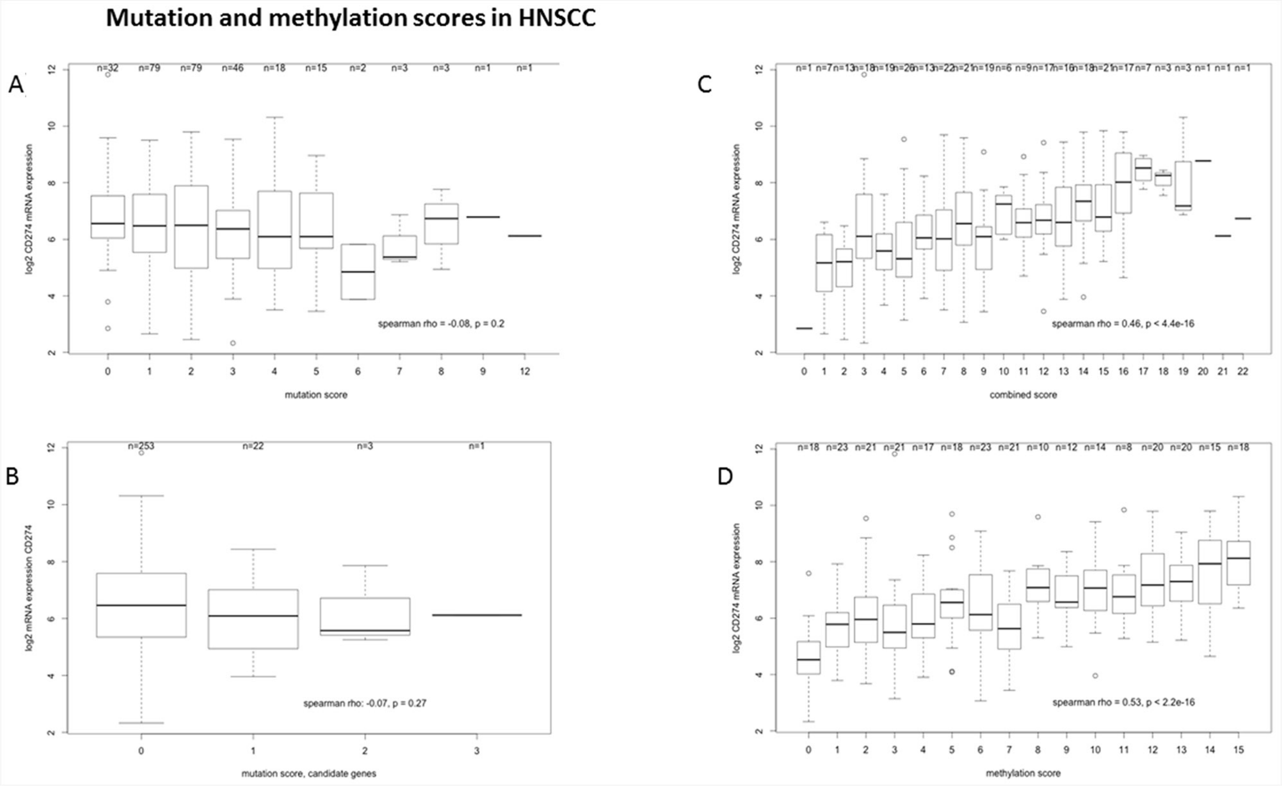

Supplementary Figure S8: Boxplots showing the relationship between mutation of DNA repair genes and mRNA expression of CD274. A. No correlation was found between DNA repair gene mutation score (+1 for every DNA repair gene mutated among all DNA repair genes, compare S2) and CD274 expression (spearman rho=-0.08, p=0.2). **B.** Similarly, no correlation was identified between a DNA gene mutation score and CD274 expression, when only DNA repair genes from the HNSCC candidate gene list were included (rho=-0.07, p=0.27). **C.** The combined score of DNA repair gene mutation (all DNA repair genes) and HNSCC candidate gene list hypermethylation (+1 for every gene with methylation greater than mean methylation for this gene) exhibits positive correlation with CD274 mRNA expression (rho=0.46, p=4.4e-16). **D.** The methylation score (+1 for every gene with methylation greater than mean methylation for this gene) shows positive correlation with logCD274 mRNA expression and performs better than the combined score (rho=0.53, p<2.2e-16).

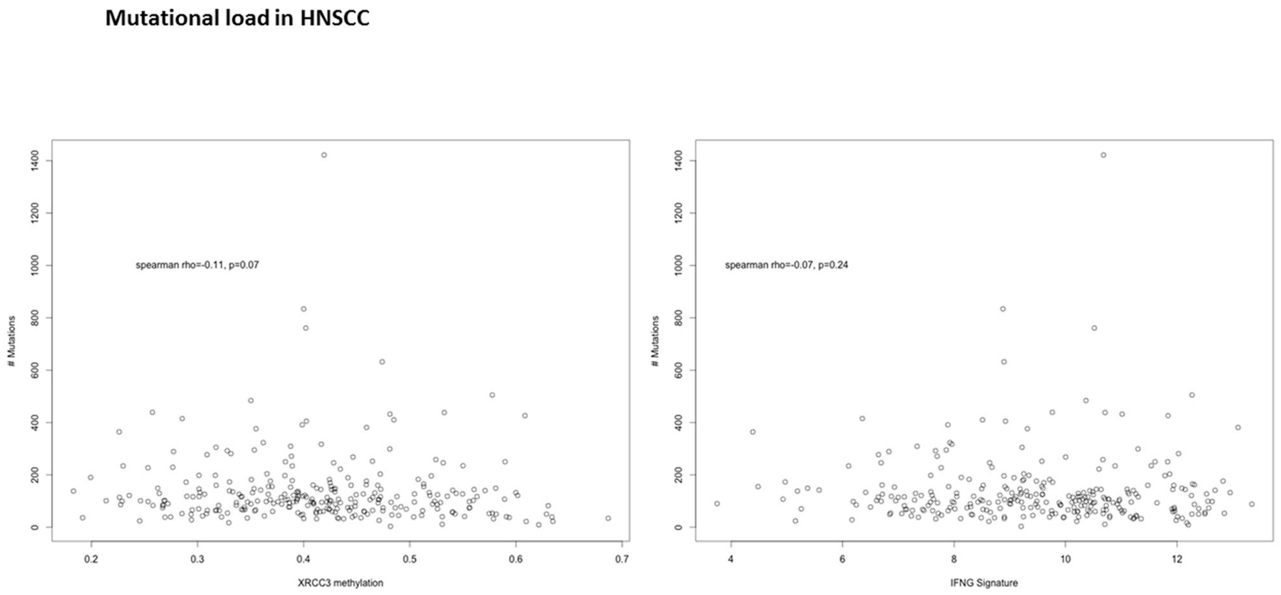

Supplementary Figure S9: Association of mutational load with DNA repair gene methylation, mutation and the IFNG signature. Mutational load is neither correlated with the IFNG signature (rho=-0.11, p=0.07), nor with XRCC3 methylation (rho=-0.07, p=0.24).

A

DNA repair gene mutations in HNSCC - 1

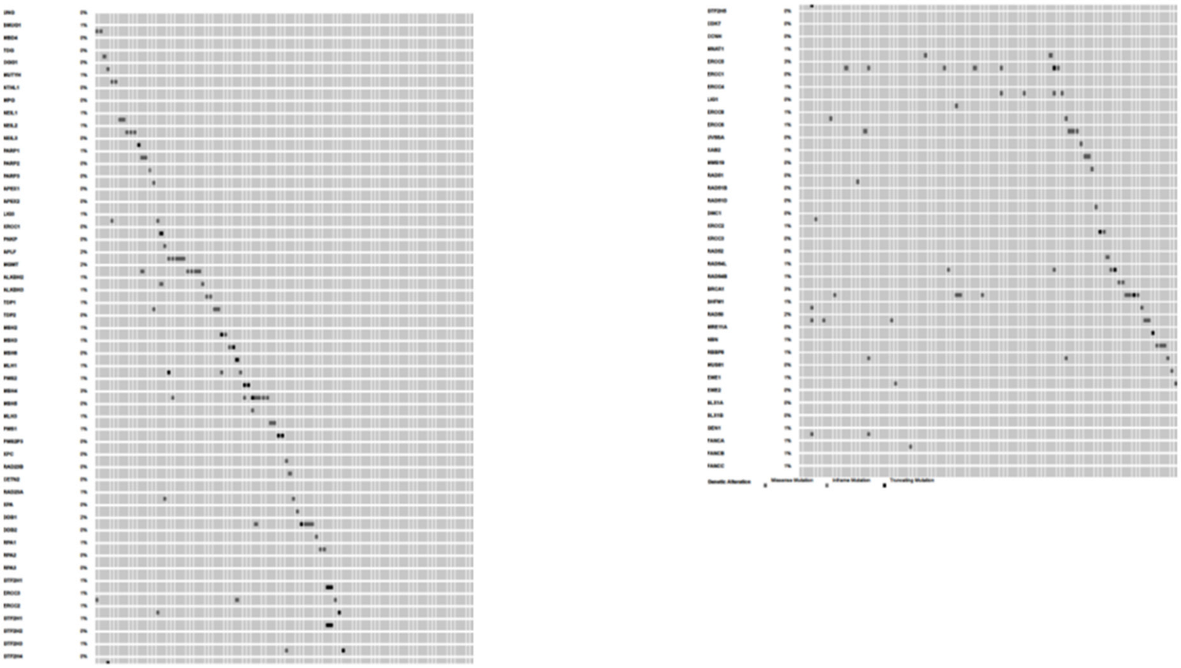

B

DNA repair gene mutations in HNSCC - 2

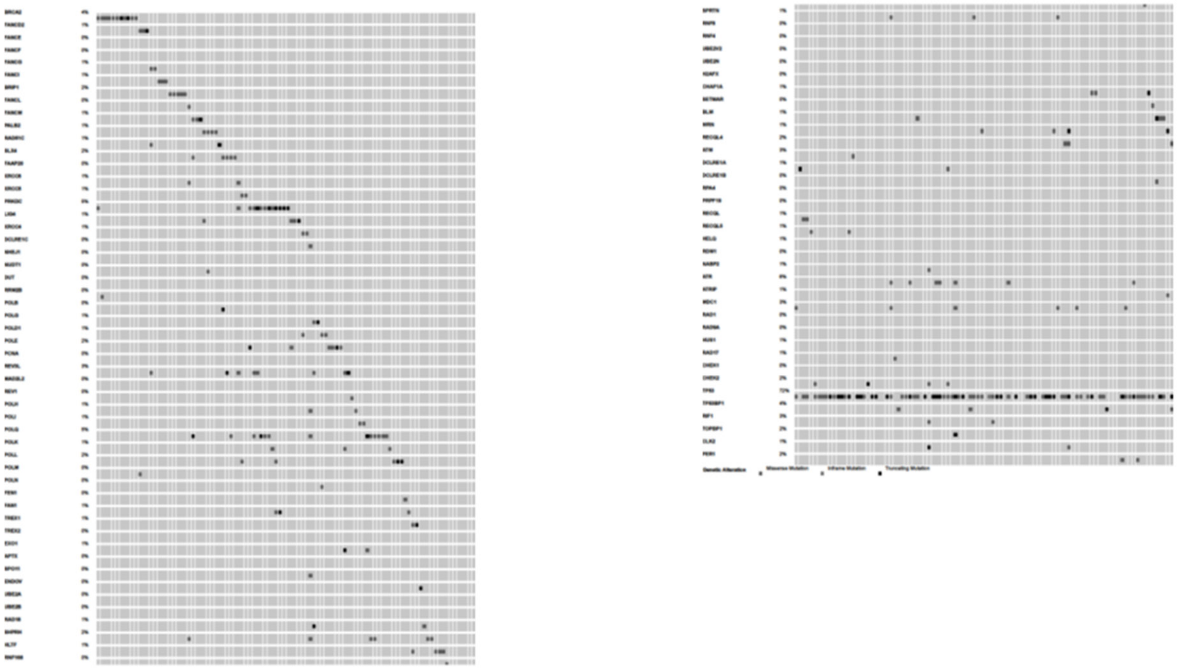

Supplementary Figure S10: A. List of DNA repair genes and prevalence of mutations in TCGA data. **B.** continued list (as derived from cbioportal.org)

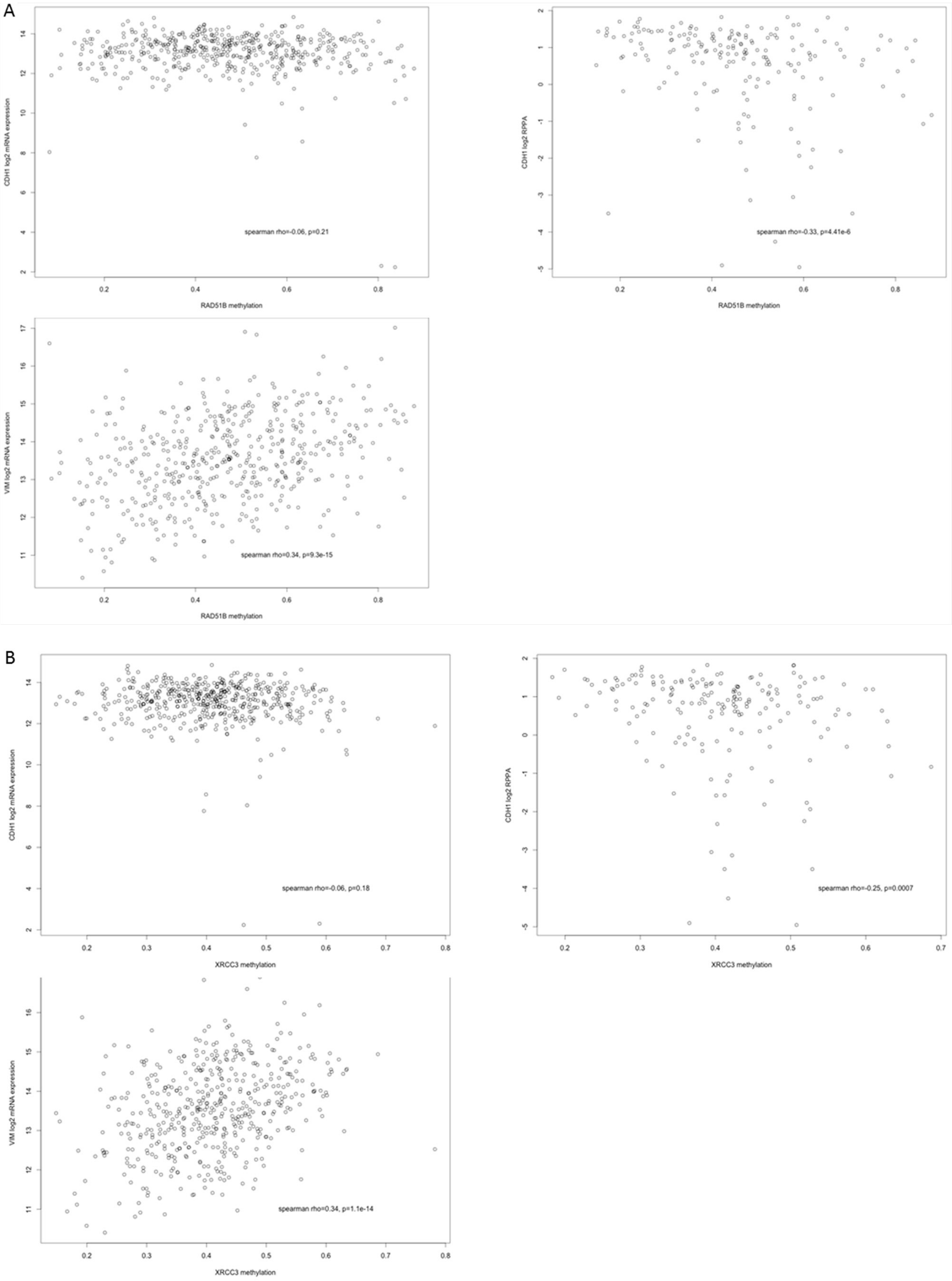

Supplementary Figure S11: Association between hypermethylation of DNA repair candidate genes and markers of an epithelial-to-mesenchymal transition in HNSCC. CDH1 indicates E-Cadherin, an epithelial marker and VIM indicates Vimentin, a mesenchymal marker. Protein (RPPA) and mRNA data are available for CDH1 **A.** RAD51B is nonsignificantly associated with reduced E-Cadherin (CDH1) mRNA expression (r=-0.06, p=0.21) but shows a statistically significant negative correlation with CDH1 protein expression (RPPA, r=-0.33, p=4.41e-06). RAD51B is furthermore statistically significantly associated with increased Vimentin (VIM) mRNA expression (r=0.34, p=9.3e-15). **B.** Similar associations are identified for XRCC3 methylation (CDH1 mRNA: rho=-0.06, p=0.18, VIM mRNA rho=0.34, p=1.1e-14, CDH1 protein expression rho=-0.25, p=0.0007).

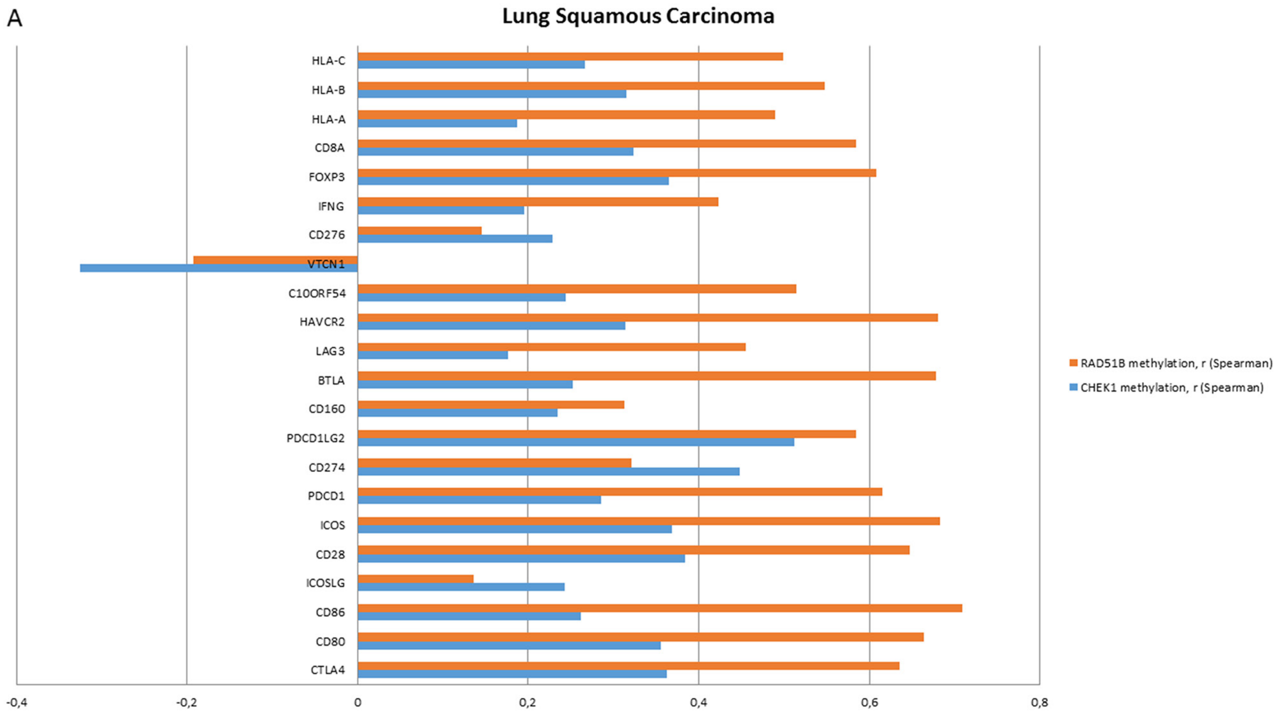

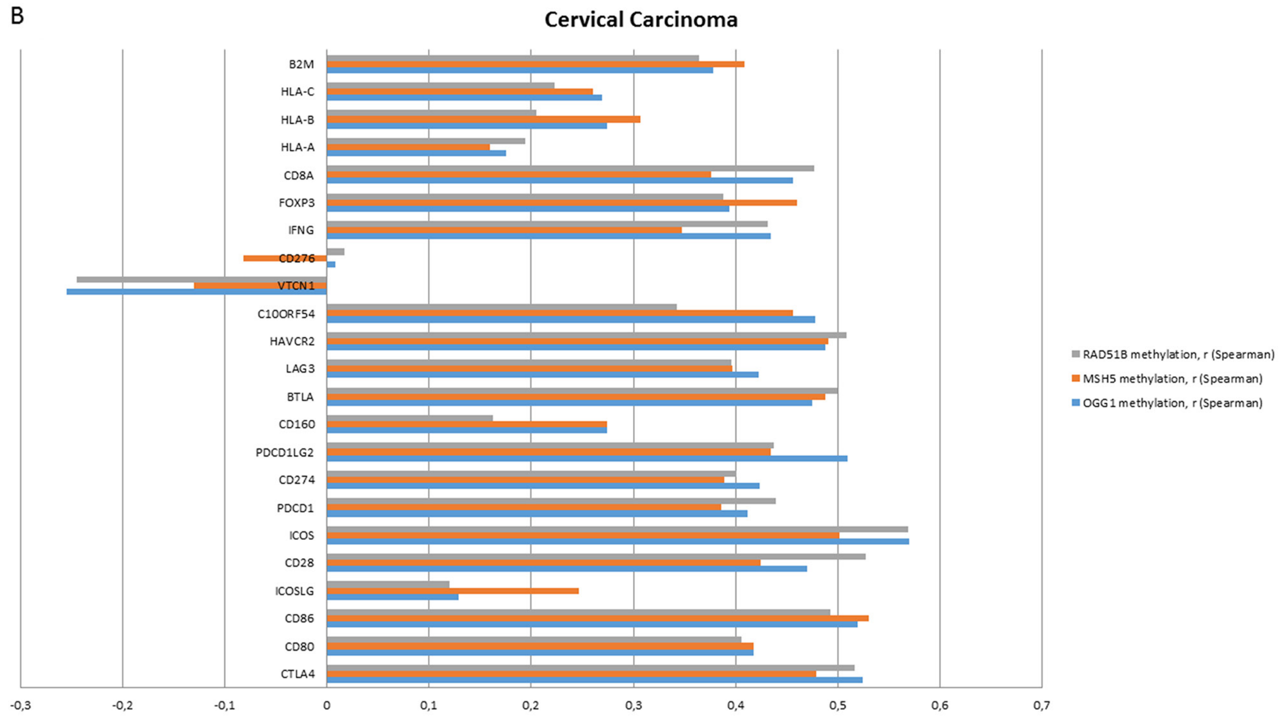

Supplementary Figure S12: Methylation of the candidate genes from lung and cervical carcinoma is analyzed for correlation with mRNA expression of a broad range of inflammation-associated genes (including checkpoint molecules, ligands, T-cell markers, interferon gamma, MHC1 and 2, TCGA data). A. Both candidate DNA repair genes (RAD51B, CHEK1) correlate with the expression of respective genes. **B.** The same is found with the cervical carcinoma candidate genes (RAD51B, MSH5, and OGG1). Here the negative correlation of VTCN1 and OGG1 is statistically significant after Bonferroni correction (p=0.009).
